# Migrant Middle Eastern women with gestational diabetes seven years after delivery – positive long-term development of beliefs about health and illness shown in follow-up interviews

**DOI:** 10.1017/S1463423621000232

**Published:** 2021-05-26

**Authors:** Katarina Hjelm, Karin Bard, Jan Apelqvist

**Affiliations:** 1 Department of Public Health and Caring Sciences, Uppsala University, Uppsala, Sweden; 2 Department of Endocrinology, Division of Diabetes Care, Malmö University Hospital, Lund University, Malmö, Sweden

**Keywords:** beliefs about health/illness/health care, gestational diabetes, migrants/Middle East, prospective study, qualitative study, self-care, semi-structured interviews

## Abstract

**Aim:**

No previous studies have been found focusing on the long-term development of beliefs about health, illness and healthcare in migrant women with gestational diabetes mellitus (GDM). The aim of this study was to explore this and the influence on health-related behaviour (i.e. self-care and care seeking) in migrant women from the Middle East living in Sweden seven years after delivery.

**Background:**

GDM is increasing, particularly in migrant women. The risk of adverse outcomes of GDM for health can be improved by interventions reducing blood glucose and lifestyle modifications which medicalise the woman’s pregnancy due to intensive follow-up and demanding self-care. The reactions might have an enduring impact on the women’s long-term psychological and physical health and adoption of preventive health behaviours.

**Method:**

Qualitative exploratory study. Semi-structured follow-up interviews 7 years after delivery with women previously interviewed in gestational weeks 34–38 and 3 and 14 months after delivery. Data analysed with qualitative content analysis.

**Findings:**

Health meant freedom from illness, feeling well and living long to be able to care for the children. The present situation was described either positively, changing to a healthier lifestyle, or negatively, with worries about being affected by type 2 diabetes. Beliefs changed among the majority of women, leading to a healthier lifestyle, and they looked positively back at the time when diagnosed and their reactions to it. With few exceptions, they were confident of being aware of future health risks and felt responsible for their own and their children’s health/lifestyle. None except those diagnosed with type 2 diabetes had been in contact with healthcare since the last follow-up a year after delivery. Yet, they still would like and need a healthcare model delivering more information, particularly on developing a healthy lifestyle for children, and with regular check-ups also after the first year after delivery.

## Introduction

Gestational diabetes mellitus (GDM) affects between 1% and 25% of pregnancies globally and its incidence is increasing (Zhu and Zhang, 2016), and migrant women are particularly affected (Hedderson *et al.*, 2012). The risk of adverse outcomes of GDM for the health of mother and child can be improved by interventions reducing blood glucose during pregnancy and lifestyle modification during pregnancy and afterwards (Paulweber *et al.*, 2010). The impact of these interventions changes the context of a woman’s pregnancy to something highly medicalised due to intensive follow-up and demanding self-care measures (Parsons *et al.*, 2018). The adoption of preventive health behaviours may be influenced by women’s experiences of GDM (Devsam *et al.*, 2013). The reactions have been described as sometimes having an enduring impact to the potential detriment of women’s long-term psychological and physical health (Parsons *et al.*, 2018; Craig *et al.*, 2020), but no previous studies have been found focusing on the long-term development of beliefs about health, illness and healthcare.

The rapid increase of GDM in migrant women in developed countries relates to ethnic origin and rising rates of obesity due to lifestyle change (Hedderson *et al.*, 2012; Carolan *et al.*, 2013a). Women with GDM have an increased risk of further episodes of GDM and a potential risk of developing glucose intolerance and type 2 diabetes in the future (Damm *et al.*, 2016). Hyperglycaemia during pregnancy has a programming effect on the long-term metabolic health of the offspring, increasing its risk of type 2 diabetes (Dabelea *et al.*, 2000; Krishnaveni *et al.*, 2010). The condition is described as a demanding and often distressing experience due to the lifestyle changes needed, emotional reactions because of the management and the perception that it might be life threatening (Jirojwong *et al.*, 2017; Parsons *et al.*, 2018; Craig *et al.*, 2020). Migrant women reported it as an unanticipated disruption of normal lifestyle (Jirojwong *et al.*, 2017), an additional burden on their life situation (Siad *et al.*, 2018), and expressed a lack of knowledge. Reactions to the diagnosis differ from shock to none at all, and thus some women describe an adaptation to the condition and feelings of responsibility for the foetus, giving motivation to follow advice from healthcare staff to manage themselves properly, while others choose denial and perceive it as invisible (Carolan, 2013b; Devsam *et al.*, 2013; Jirojwong *et al.*, 2017; Siad *et al.*, 2018; Parsons *et al.*, 2018).

There is extensive ongoing global migration, and many of the recent immigrants originate from the Middle East (IOM, 2020; SCB, 2016). Individual beliefs about health and illness are culturally determined, constituting attitudes guiding health-related behaviour (Glantz *et al.*, 2015; Rosenstock *et al.*, 1988). This consequently influences healthcare seeking, preventive self-care and health (Hjelm *et al.*, 2003; 2013; 2012a,b; 2018). Knowledge about beliefs is important in work to influence various risk factors to promote health and prevent type 2 diabetes in both mother and child.

There is extensive ongoing global migration, and many of the recent immigrants originate from the Middle East (IOM, 2020; SCB, 2016). Individual beliefs about health and illness are culturally determined, constituting attitudes guiding health-related behaviour (Glantz *et al.*, 2015; Rosenstock *et al.*, 1988) influencing healthcare seeking, preventive self-care and health (Hjelm *et al.*, 2003; 2012a,b; 2013; 2018). Knowledge about beliefs is important in work to influence various risk factors to promote health and prevent type 2 diabetes in both mother and child.

A previous study on beliefs about health and illness showed that pregnant migrant women with GDM from the Middle East perceived GDM as a transient condition with unknown cause, had a passive attitude to self-care and perceived pregnancy-related complications as natural, in contrast to Swedish women who saw GDM as a risk marker for type 2 diabetes and feared its development (Hjelm *et al.*, 2005). When evaluating care for these women, it was found that the healthcare model did not promote active information-seeking and active participation in self-care (Hjelm *et al*., 2007). However, the question is what happens in the new country when the individual is influenced both by the lifestyle and habits in the new country and those brought from the home country (Berry, 2017). Are individual beliefs about health and illness changed over time, and are thus lifestyle and health-related behaviour influenced? How is the contact with healthcare perceived, what impact do educational activities have, and what support is needed over time, from during pregnancy, after delivery and later in life?

Previous studies of temporal development of beliefs about health and illness in migrant women with GDM are restricted to two investigations with a limited follow-up time of 14 months (Hjelm *et al.*, 2012a; 2018), focusing on women born in the Middle East and Africa living in Sweden. An U-shaped curve was shown in Middle Eastern women (Hjelm *et al.*, 2012a) with regression after delivery to a less healthy lifestyle (poorer dietary habits, less exercise) than before diagnosis and shift in focus from trying to adhere to given advice and worrying about the baby’s health during pregnancy to their own risk of developing type 2 diabetes and fear of being unable to care for the child. This was in contrast to a linear course with unchanged low risk awareness and limited knowledge prospectively in African women (Hjelm *et al.*, 2018). However, in the literature search, no previous studies have been found examining the long-term development of beliefs about health, illness and healthcare in migrant women with GDM. The aim of this study was to explore this and the influence on health-related behaviour (i.e. self-care and care seeking) in migrant women from the Middle East living in Sweden seven years after delivery.

## Methods

### Design

A qualitative exploratory study design was applied (Patton, 2015). Individual semi-structured follow-up interviews were held 7 years after delivery with women previously interviewed in gestational weeks 34–38 and 3 and 14 months after delivery. The interview style encouraged respondents to tell their stories freely, to give a deeper understanding of their perceptions (Patton, 2015).

### Management of gestational diabetes

Women in this study were met seven years after delivery. Initially, they had been screened for GDM in a healthcare centre by a midwife (28th or 12th gestational week) and then referred to a diabetes care team at a specialist diabetes clinic (Hjelm *et al.*, 2012a). Additional diagnostic tests were taken, and 1–2 weeks later information about GDM was given by a diabetologist, whereafter regular personal contacts with a diabetologist or diabetes specialist nurse took place every third week during pregnancy in uncomplicated cases or individually prescribed. Self-monitoring of blood glucose was prescribed daily in the case of insulin treatment, otherwise every second day until delivery. Eight weeks postpartum, there was a planned visit to a midwife and/or an obstetrician for family planning and contact with a diabetologist in case of continued testing after delivery. Finally, about a year postpartum, to a diabetes specialist nurse for oral glucose tolerance test and information about the importance of exercise and weight reduction. Finally, the last planned visit was about a year postpartum to a diabetes specialist nurse for oral glucose tolerance test and information about the importance of exercise and weight reduction.

### Participants

Initially, fourteen women born in countries in the Middle East, diagnosed with GDM and living in Sweden, had been consecutively recruited by staff at a hospital-based diabetes specialist clinic to participate in the prospective study. In the last interview, 14 months after delivery, the women had consented to be contacted for follow-up. Four of them did not attend as they failed to respond, had moved to unknown addresses abroad (n = 2) or other parts of the country, leaving nine women for inclusion.

### Data collection

A semi-structured interview guide with open-ended questions was used. Initially, standardised questions on medical and socio-demographic data were posed. The interview guide was developed based on a previous study (Hjelm *et al.*, 2012a), review of the literature and peer-review by diabetes specialist nurses, midwives and diabetologists managing women with GDM. Themes explored are presented in Table 1. The interview had a retrospective perspective with a focus on reflections on how to maintain health and were held by an external specialist nurse (second author) not involved in the management of the women or in the studied clinic. An authorised female interpreter was used, and the sequential interpretation technique with interpreting word for word was applied as recommended in Sweden (Hadziabdic & Hjelm, 2013; Kammarkollegiet, 2016). The interviews were held outside the clinic, in secluded rooms, lasted about 1.5 h, were recorded and transcribed literally. The texts were coherent and without non-translated parts.

**Table 1.** Themes explored in the semi-structured interviews

Beliefs about health, illness and healthcare, including perceptions of:
– the present situation and compared to when being diagnosed with GDM
– factors of importance for health,
– future health
– explanations and consequences of GDM
– care-seeking behaviour
– management since last interview 14 months after delivery
– need of support
– self-care advice
– self-reported adherence to advice received

### Ethical considerations

The study was carried out with written informed consent from the respondents and in accordance with the Helsinki Declaration (World Medical Association 2013) and approved by the Ethics Committee of Linköping University. During the interviews, the interviewer was observant of signs of distress in the respondents, who could, if judged necessary, be referred to a social worker or a psychologist, but no problems arose.

### Data analysis

Collection and analysis of data proceeded simultaneously until no new information was added in the analysis by new informants (Patton, 2015). Qualitative content analysis was performed and the aim was to be open for as much variation as possible in the material, and searching for overarching patterns and contradictions by comparing the informants’ statements. By reviewing each line of the text, topics were identified, and then the material was extracted and condensed into categories. Categories were thus developed inductively given labels as close to the original text as possible. Also introduced to the data were categories from theoretical models, such as the lay theory of illness causation by Helman (2007) and the model of health-care seeking behaviour by Kleinman (1980), for further details see Hjelm *et al.* (2012a). Example of the data analysis see Table 2. The tapes were listened through and notes were made after each interview on general findings, arising ideas and themes.

**Table 2.** Beliefs about health

Main Analytical Category ^a^	Subcategory	Quotation
Individual factors	Freedom from illness	‘Health means feeling well…the body is fit…having a good life” (R7:8)
	Feeling well	
	Wellbeing and active lifestyle with healthy diet and exercise	“…great changes in my life…lost weight…better confidence, I am very active…not tired as before…I do a lot of activities with my children”. (R7:1)
	Developing an active strategy, thinking positively	“I am positive in myself…try to avoid stress…try to think of things that give me joy…meet people…” (R7:5)
Social factors	Having a passive strategy, relying on support from others	“To get support from others to manage this situation” (R 7:2)
	Teaching children a healthy life-style and getting advice for developing a healthy life-style for them	“I have to strive…exercise and eat healthy…I try to prevent…talk with my children and give them advice about food…regular meal times…asking for advice about healthy food and life for my children” (R7:3)
Individual and social factors	Living long for the sake of the children by taking responsibility for own health and managing diseases	“Being able to live long… I don’t bother about diseases or if I am careless it will cause troubles for me and my children…”(R7:9)

a Main analytical categories from the lay model of illness causation by Helman (2007) where causes of illness could be related to the individual, nature, social relations and/or the supernatural world.

By including two persons in the analysis of data, a diabetes specialist nurse and a general nurse (first and second author), the trustworthiness of the findings was increased (Patton, 2015). The first author checked the content of the categorised data and high agreement was shown. Certified and specially trained interpreters, using a sequential interpretation technique, were used to decrease influence of language problems related to interpretation (Hadziabdic & Hjelm, 2013). To compensate for second-order interpretations of the foreign culture studied (Triandis, 2000), culture-specific phenomena and expressions were discussed with the interpreters, who had a profound knowledge of the culture. Results are presented as categories with illuminating quotations to achieve confirmability (Patton, 2015). Dependability is ensured by a detailed description of each part of the research. Results from qualitative studies are transferable to other persons and contexts similar in characteristics, when carefully handled and described.

## Findings

### Beliefs about health

Health seven years after delivery was described as being free from illness, feeling well and living long for the sake of the children by taking responsibility for one’s own health and managing diseases in the best possible way (individual and social factors; Table 2):

Health means feeling well…the body is fit…having a good life. (R7:8)

Being able to live long…a longer time to see my children…if I don’t bother about diseases or if I am careless it will cause troubles for me and my children… (R 7:9)

When describing the current situation the respondents expressed it either in a favourable way, with positive changes, particularly the lifestyle and feeling well, or in a negative way, with some feeling worse than before due to worries about the genetic risk of developing type 2 diabetes, being diagnosed with diabetes, or feeling tired during pregnancy.

…great changes in my life…lost weight…better confidence, I am very active…not tired as before…before I was lazy…now I do a lot of activities with my children. (R7:1)

…always tired…no appetite…put on weight…worried about increased blood glucose…It feels like something inherited…I have tried to exercise…I act as if I am worried about getting or having developed. (R7:3).

Compared to their lifestyle during pregnancy, the women told they now lived as during pregnancy, or as before pregnancy, or that they had changed to a lifestyle with more exercise and healthier diet.

In order to feel well and experience good health, some had an active strategy, thinking positively, avoiding negative thoughts and stress, and trying to do everything they could cope with, while others were passive or relying on support from others.

…try to avoid stress,…I try to think of things that give me joy…meet people, to have a lot of people around me. (R7:5)

Nothing particular. (R 7:4)

To get support from others to manage this situation. (R7:2)

Individual factors, such as mental well-being and an active lifestyle with exercise and a healthy diet, and social factors such as teaching the children a healthy lifestyle and getting advice for developing this for them and trying to live as long as possible for them were stated as important for health when diagnosed with GDM.

…beauty of the soul…ill-health also influences your social life…I…have to strive…exercise and eat healthy…I try to prevent…talk with my children and give them advice… (R 7:3)

It doesn’t matter how long diabetes persists or if I am affected by other diseases, I think about my children in the first place. (R7:9)

With few exceptions, tangible support was perceived as important to relieve pressures, being reminded of healthy habits, and being cheered up, all of which promoted health. Health professionals were seen as important for health by being engaged in their situation, giving advice and follow-ups, and one person added their significance in being able to have a holistic view of their situation.

…they (health professionals) help and support me at home. Ask if I have taken my medicine and remind me… (R 7:9)

…without them I would not get information and help… (R7:4)

Important ways to maintain health and prevent complications were lifestyle factors (individual factors) such as diet, exercise and trying to decrease stress and avoid negative thoughts, although some said they did ‘nothing’. For the majority (some were believing Muslims) it was important to have a religious belief and to pray (supernatural factors) while others said it was of ‘no importance’. None, except one, regarded celebrating feasts and traditions as important for health. Nature cure remedies (e.g. herbs, cinnamon, vitamins, Omega 3) were used by half of the respondents. Economy was perceived as important for health to the same extent. Diet for persons with diabetes was reported to be more expensive than ordinary food and thus, they were unable to buy what they ought. Visits to physicians and medications were also seen as expensive.

Diet, much exercise, less stress. (R7:5)

I try with my thoughts, not thinking in a negative way, to be left in peace. (R 7:7)

…we have a religion that instructs us to eat just enough, exercise a lot…just eat when you are hungry…no more than needed. (R7:1)

I do not have any problems with religion, it does not affect my health. (R 7:2)

It (economy) has a big influence as when shopping you do not buy the same food for the others in the family…you have to buy what is appropriate for a person with diabetes. And you have to exercise and that costs too. So some things that are good for a person with diabetes I do not buy…it is food…vegetables…so just don’t buy them. (R7:9)

A diet rich in fibres was seen as the most appropriate seven years after delivery. Some also said that their diet was reduced in sugar and fat and rich in proteins. This was in accordance with advice given at the last visit a year after delivery, which they all claim to follow.

All, except one, stated walking as appropriate exercise and most had received advice about exercise and followed it. A few took exercise by swimming and running. None had been advised about self-monitoring of blood glucose (SMBG) or medications at the last visit to healthcare. Only one person (diagnosed with diabetes) still did SMBG and another wished she could have check-ups.

### Beliefs about illness

Most did not experience any health problems related to GDM after pregnancy since the last interview 14 months after delivery. When looking back on when diagnosed with GDM, the majority today viewed the condition positively and were no longer worried but instead felt secure and aware of their own responsibility for lifestyle and the risk of relapse.

Now I am more aware and have more knowledge about it (GDM) than before, at the beginning I didn’t know anything and became very worried and thought it was dangerous…Now I think that if I get diabetes I know how to deal with it and…how to change my living habits…first diet…eating meals regularly, at set times…getting exercise. (7:4).

When discussing their own perception of the current and future health of the child, the majority feel that self-care and good health are important, but some worry more about the health of the child, while one replied ‘don’t know’ and told she relied on God but tried to teach the children healthy dietary habits. Thus, most focus on factors related to the individual but this woman also mixed supernatural thoughts. When discussing the consequences of GDM for future health, most expressed worries about an increased risk of future diabetes, while one spoke about her own risk due to focusing on the health of the children and neglecting her own needs and health, and some did not see any consequences at all.

I have to keep on with exercising and diet. It is the most important. I nag my children to eat a lot of vegetables and such…I eat a lot of vegetables…my husband says they can eat whatever they want…Then I become worried, not only for my children. (R 7:1)

I think a lot about risks…it is frightening to think that my children will be affected (by diabetes)…as adults have difficulties with it. (R 7:3)

I don’t know…I rely on God…I also try to teach them and give them advice and tips about food and set times for meals. (R 7:4)

I do not have time to bother about myself…I do not feel well but I do not have space for it…I have a tendency to neglect everything that has to do with my health. But when it comes to the children I take care that they get everything they need…their father too… (R7:2)

The majority perceive GDM as persisting forever, a few say that they ‘don’t know’ if it is still there and a couple that it is ‘gone’. Most fear developing diabetes or complications, whereas some does not fear anything. For most women the main problems that GDM have caused were psychological, such as worries about health and about living conditions, such as residence permits etc., which are thought to cause the condition. However, some did not experience any problems.

I have read about diabetes and maybe problems with the kidneys…leg or foot amputated…don’t feel when you get a wound…in the foot. So that scares me…eye complications. (R 7:4)

No I do not fear anything. You have so much else to think about so you do not think about it. (R 7:6)

### Beliefs about healthcare

With few exceptions, most had not been followed-up since the last interview (14 months after delivery) and they felt a lack of regular check-ups. One person had been diagnosed with type 2 diabetes and thus had been checked regularly. Most had not experienced any influence of pregnancy on their health except worries about the risk of the child getting diabetes.

During pregnancy…contact with health care once a week…until delivery, then it was not until a year after I met them again…After pregnancy there were no follow-ups.

Interviewer: Do you miss that?

Yes. (R7:4)

No contact…wish that I could be contacted once a year. (R7:5)

No, nothing. But I have been worrying about my children getting it (diabetes). (R 7:1)

With two exceptions, none needed to seek care since the last interview, one had felt ill and diabetes was diagnosed at the healthcare centre and another had gone to the emergency department with nephrolithiasis. However, about half of the respondents experience insufficient support during the time since the last check-up at 14 months after delivery and desire regular check-ups. They told they were well controlled during pregnancy but not afterwards when they feel neglected and left without anyone bothering about them.

Three times a week…I test (blood glucose), both fasting and after having eaten…They send letters for follow-ups…the diabetes specialist nurse follows me…the physician will see me after three months. (R 7:9; relapsed patient)

During the time (pregnancy) but when you have been affected with something like this you ought to be checked at least once a year or every second year but not like here, one or two times and then nothing…I lack that…feel denied. (R7:3)

All except one claim they do not get any support from health care, lack it and want to be checked up, get advice and medications. Further, they experience problems accessing the ‘right doctor’ who can understand their problems, and lack the time to manage themselves, e.g. with exercise.

When needing help, most turn to the professional healthcare sector, in the first instance the healthcare centre and then the emergency department. However, a couple of women do not know where to turn. The accessibility of healthcare is mainly regarded as good, but for some it is difficult to get an appointment with a physician; most are content with the contact with staff although some mention language barriers. In summary, about half of the respondents feel that they get the help they need while the rest say that it can be difficult without an interpreter.

It is easy, I am very content. (R7:1)

It is difficult to phone and…to get time for an appointment…when I finally manage to reach them they say the timetable for the week is full and I need to phone again tomorrow at 8 or 9 when they have telephone time and then you maybe wait half an hour or sometimes more, they say, unfortunately it is finished! (R7:4)

Sometimes I feel I need an interpreter, sometimes I manage on my own but in some situations it’s difficult… (R7:4)

The respondents expect to meet competent healthcare staff, working fast and effectively, and the majority do not experience any problem in contact with them. The majority perceive the care given during pregnancy as good but wish for more advice, information and continuous regular check-ups given by the ideal nurse or physician who is a good listener that gives help and support and provides regular check-ups.

…that they are competent…that I get the help needed…at once and do not need to wait for months to see a doctor. (R 7:5)

More check-ups…regularly…cared a lot during pregnancy…up to delivery…I got frequent appointments, I got advice…it was good…but as soon as the delivery was over it felt as their commission was ended…then they didn’t bother about the mother. (R7:2)

Advice on how to eat, how to live healthy, what to do to manage yourself. Not just blood tests and then go away. They should do things like that also…I haven’t got that. (R 7:3)

## Discussion

This is the first study investigating the long-term development of beliefs about health, illness and healthcare in migrant Middle Eastern-born women with GDM seven years after delivery. The main findings showed that health meant freedom from illness, feeling well and being able to live long to care for the children. The present situation was described either in a positive way, with changes to a healthier lifestyle, or in a negative way, with worries about the risk of being affected by type 2 diabetes. The majority look back positively at the time when diagnosed and their reactions to it, and feel secure as they are aware of future health risks and know they are responsible for their own and their children’s health and lifestyle. They have changed their lifestyle and would like more information, particularly to develop healthy habits for the children. None, except those diagnosed type 2 diabetes, have had contact with healthcare since the last follow-up a year after delivery, focusing solely on dietary advice and exercise. The care delivered during pregnancy and up to that point was perceived as well-functioning but more information and regular check-ups after the first year and onwards were requested.

As in the previous study during pregnancy (Hjelm *et al*., 2005) and in the follow-ups 3 and 14 months after delivery (Hjelm *et al.*, 2012a), the focus in health beliefs was still on freedom from illness, well-being, worries about the baby’s health and being able to live long to care for the children. The women reported adhering to previously received advice from healthcare staff (during pregnancy, reminder a year after delivery) and most said they had developed an active health behaviour with an even healthier lifestyle but differed from the previous investigations as respondents focused on healthy habits for the children and themselves and not only their own worries about the risk of developing type 2 diabetes and being unable to bring up their children. They wished to learn about healthy habits for the child. Thus, most had a positive view of their present situation while some still were negative due to worries about their own health risks, and an active problem-solving coping strategy seemed to dominate over a passive emotion-focused one where women did not take an active part (Folkman, 1984) in self-care as during pregnancy (Hjelm *et al.*, 2005). As previously described, the reactions to the diagnosis differ (Devsam *et al.*, 2013; Jirojwong *et al.*, 2017; Siad *et al.*, 2018; Parsons *et al.*, 2018), and an adaptation to the condition is needed to cope with the situation and maintain health (Folkman, 1984). A change during pregnancy (although not studied after it) has previously been described, with an increased feeling of confidence in effectively managing the condition and a sense of empowerment and self-efficacy, gradually finding a balance as the women became familiar with GDM (Jirojwong *et al.*, 2017) and could cope with the increased stress as the condition became part of their daily routine (Persson *et al.*, 2010). The coping strategy might be influenced by the potential negative impact on women’s long-term emotional and physical health caused by the reactions to the diagnosis and experiences of the management (Parsons *et al.*, 2018). Other influencing factors might be knowledge and educational level influencing perceived locus of control, which seem to mainly be internal (Rotter, 1996) as the majority described being active in self-care (Rosenstock *et al.*, 1988) and low level of knowledge is related to passive health behaviour due to low-risk awareness (Bandura, 1995). Also migrational background/experiences, adaptational problems in the new country (Berry, 2017) and other socioeconomic factors exert an influence (Rosenstock *et al.*, 1988). GDM has been perceived as an additional burden on an already strained life situation in migrants (Siad *et al.*, 2018).

The adoption of preventive health behaviours may be influenced by women’s experiences of GDM (Devsam *et al.*, 2013), and in this study, the majority of the respondents looked back on the time of being diagnosed (Hjelm *et al.*, 2005) and the reactions to it in a positive way and felt confident in their awareness of future health risks and the knowledge that they are responsible for and can influence their own and their children’s health and lifestyle. Compared to the results of the previous studies with interviews from during pregnancy (Hjelm *et al.*, 2005) and onwards (3 and 14 months after delivery) (Hjelm *et al.*, 2012a) increased health awareness has developed and the health message that type 2 diabetes can be prevented by an active lifestyle and healthy diet (Paulweber *et al.*, 2010; Uusitupa *et al.*, 2018) seems to have been adopted by most of the women. With few exceptions they also report having developed a realistic risk awareness of being affected with GDM and describe the importance of attending to their own health needs despite living in a demanding situation, being preoccupied with their children and having limited time for self-care. However, some still perceived the condition as transient, as during pregnancy (Hjelm *et al.*, 2005), without any consequences and thus followed a passive self-care behaviour or worried about the future. It is therefore important to identify individual beliefs about health and illness and the perceived seriousness of the condition early and work with (Hjelm *et al.*, 2012a; 2018). This was further supported as neither this nor previous studies (Hjelm *et al.*, 2003; 2005; 2012a,b; 2018) confirmed the hypothesised dominance of beliefs related to supernatural factors (Helman, 2007) and use of folk medicine (Kleinman, 1980) despite the majority being believing Muslims and of non-European origin.

Since the last follow-up a year after delivery, none of the respondents have had contact with healthcare except those diagnosed with type 2 diabetes (n = 2). As in the previous studies (Hjelm *et al.*, 2007; 2012a), the care delivered during pregnancy and the first year is experienced working well but still they lack more information at the last follow-up, would like regular follow-ups, and more information particularly about how to develop healthy habits for their children. It is evident that GDM and the risk of developing type 2 diabetes is a public health concern (Paulweber *et al.*, 2010). The respondents’ call for information needs to be heeded, and their willingness to adhere to recommendations is a positive indicator that they will carry on with measures to prevent type 2 diabetes and promote health.

The risk of presenting with GDM and postpartum diabetes has increased over the years (30% at 5 years, 51% abnormal glucose tolerance) (Ekelund *et al.*, 2010), particularly in migrants of non-European origin (17% vs European 4%), although more strongly related to obesity (Ignell *et al.*, 2013) developing in many migrant women. Thus, the number of affected migrant women in the studied clinic has increased (personal communication), further aggravated by new diagnostic criteria for GDM (WHO 2013). The clinic is presently involved in a national evaluation of the outcome of these (Fadl *et al.*, 2019) aimed to fill the gap in common national guidelines for screening and management of GDM. However, the routine has changed slightly since the last follow-up a year after diagnosis. Initial information is given by a midwife, instead of a diabetologist, focusing on achieving good glycaemic control during pregnancy and everyone, not only those deemed in need, is offered contact with a dietician for advice.

### Implications

The results show different beliefs and health-related behaviours and a need for more knowledge both for those requesting information to further develop a healthy lifestyle, particularly for their children, and for those who lack adequate knowledge about the seriousness of the condition and thus neglect it or are unnecessarily worried. Interestingly, none except those diagnosed diabetes have been in contact with healthcare since the last follow-up a year after delivery and all are satisfied with the care delivered during pregnancy and the first year, but request more information and regular follow-ups afterwards. The question is if more information, delivered in a different way, should have been given or if regular-follow-ups provided during the seven years up to the present situation would have given the best results. This need to be further investigated.

The healthcare model studied neither supported their needs for information nor stimulated active participation in self-care, as previously concluded (Hjelm *et al.*, 2007). Discussions of how to develop healthy habits for mother and child and in relation to the prevention of diabetes, irrespective of having had GDM or not, could be included in parental education. Parental groups run from pregnancy onwards and are meant to continue even during childhood, and thus a raised awareness of GDM and prevention of type 2 diabetes could be taught to the general population, as recommended (Paulweber *et al.*, 2010). A population-based strategy is needed, including women yet to become pregnant and those already pregnant and their families (Simmons 2015). There is also a need for general health education in society to increase risk awareness through mass-media campaigns (Paulweber *et al.*, 2010).

There are still some who perceive the condition as transient, and thus are passive in self-care, while others are passive due to worries about the future. It is therefore important to identify individual beliefs about health and illness and the perceived seriousness of the condition early, to work with and find adequate support for coping strategies (Hjelm *et al.*, 2012a; 2018). Information about future health and health risks needs to be included in health education.

Besides the importance of developing a healthy lifestyle, with healthy diet and exercise, economic factors were also believed to influence health. Recommended diet was seen as expensive, and strained economic living conditions are a reality for many migrants (Folkhälsomyndigheten, 2018). However, a healthy diet does not need to be expensive, which also needs to be discussed in health education.

### Limitations

There is a risk of recall bias (Patton, 2015) when investigating beliefs seven years after a previous study and having another baby (*n* = 2). However, some of the results show a similar pattern to previous studies (Hjelm *et al.*, 2005; 2012a), and analysis of data show a consistent pattern. The prospective study design of the project, keeping data in context, limits the influence (Patton, 2015).

Another limitation is interviewer bias (Patton 2015), when respondents answer as they think the interviewer expects, but the interviewer in this study was unknown to the respondents, which minimised this. During the three previous interviews, during pregnancy and after delivery (3 and 14 months), the respondents had met the same interviewer (diabetes specialist nurse/researcher), which might have influenced the beliefs, and a heightened risk perception might be a consequence of the study (Glanz *et al.*, 2015). The entry of a researcher into a field of investigation will change the situation and raise awareness in those involved (Flick, 2018). Thus, lower perception of the seriousness of the disease and less knowledge are likely without this investigation.

The study population is limited due to some drop-out as some had moved to unknown addresses. However, the material showed a homogeneous picture. Carefully collected, analysed and described findings from a qualitative explorative study contribute an understanding of the studied phenomenon, make different perspectives visible and can be transferable to similar groups/contexts. Although they cannot be used for explanations or generalisations, they can help in developing hypothesis to be tested in other investigations (Patton, 2015).

## Conclusions

In conclusion, beliefs about health and illness have changed during the seven years since delivery among the majority of women studied, leading to a healthier lifestyle. They look back positively at the time when they were diagnosed and reacted to this, and with few exceptions they are aware of future health risks and knowledgeable about their responsibility for their own and their children’s health and lifestyle. Still, a healthcare model delivering more information, particularly about developing a healthy lifestyle for children, and with regular check-ups also after the first year after delivery, is requested and needed to fill in gaps in knowledge, although the initial educational support still did have an influence.
